# A clinical prediction rule to identify difficult intubation in children with Robin sequence requiring mandibular distraction osteogenesis based on craniofacial CT measures

**DOI:** 10.1186/s12871-019-0889-1

**Published:** 2019-11-21

**Authors:** Zhe Mao, Na Zhang, Yingqiu Cui

**Affiliations:** Guangzhou Women and Children’s Medical Center, No 9, Jinsui Road, Guangzhou, 510623 Guangdong China

**Keywords:** Difficult intubation, Mandibular micrognathia, Robin sequence

## Abstract

**Background:**

Airway management is challenging in children with Robin sequence (RS) requiring mandibular distraction osteogenesis (MDO). We derived and validated a prediction rule to identify difficult intubation before MDO for children with RS based on craniofacial computed tomography (CT) images.

**Method:**

This was a retrospective study of 69 children with RS requiring MDO from November 2016 to June 2018. Multiple CT imaging parameters and baseline characteristic (sex, age, gestational age, body mass index [BMI]) were compared between children with normal and difficult intubation according to Cormack−Lehane classification. A clinical prediction rule was established to identify difficult intubation using group differences in CT parameters (eleven distances, six angles, one section cross-sectional area, and three segment volumes) and clinicodemographic characteristics. Predictive accuracy was evaluated by receiver operating characteristic (ROC) curve analysis.

**Results:**

The overall incidence of difficult intubation was 56.52%, and there was no significant difference in sex ratio, age, weight, height, BMI, or gestational age between groups. The distance between the root of the tongue and posterior pharyngeal wall was significantly shorter, the bilateral mandibular angle shallower, and the cross-sectional area at the epiglottis tip smaller in the difficult intubation group. A clinical prediction rule based on airway cross-sectional area at the tip of the epiglottis was established. Area > 36.97 mm^2^ predicted difficult intubation while area < 36.97 mm^2^ predicted normal intubation with 100% sensitivity, 62.5% specificity, 78.6% positive predictive value, and 100% negative predictive value (area under the ROC curve = 0.8125).

**Conclusion:**

Computed tomography measures can objectively evaluate upper airway morphology in patients with RS for prediction of difficult intubation. If validated in a larger series, the measures identified could be incorporated into airway assessment tools to guide treatment decisions.

This was a retrospective study and was granted permission to access and use these medical records by the ethics committee of Guangzhou Women and Children’s Medical Center.

**Trials registration:**

Registration No. ChiCTR1800018252, NaZhang, Sept 7 2018.

## Background

Robin sequence (RS) is a congenital craniofacial abnormality usually defined by a triad of micrognathia, glossoptosis, and U-shaped cleft palate that collectively result in frequent tongue-based airway obstruction (TBAO). The condition affects 1 in 8500 to 20,000 neonates, and may be associated with several other syndromes [1, 2]. Most RS patients are either asymptomatic or can be treated conservatively [3]. However, patients with severe TBAO may require surgical intervention [4]. Tracheostomy is a direct and effective method to relieve upper airwway obstruction [5]. However, long-term reliance on tracheotomy can lead to bleeding, speech and swallowing difficulties, tracheal stenosis, and even death [6]. In recent years, mandibular distraction osteogenesis (MDO) has become one of the most popular surgical alternatives to tracheostomy. By gradual lengthening the mandible, thereby simultaneously advancing the soft tissues and tongue, MDO can increase upper airway size and relieve airway obstruction safely and effectively [7].

However, MDO surgery requires tracheal intubation for general anesthesia, which may be challenging in RS due to upper airway deformity. Indeed, Denise et al. reported difficult laryngoscopy exposure in 42.7% of children with RS [8] and Yin et al. reported difficult intubation in 71% of children with RS [9]. The need for more than two direct laryngoscopy attempts in children with difficult tracheal intubation is associated with high failure rate and increased incidence of severe complications, including subglottic narrowing, aspiration, and death [10, 11]. Therefore, it is critical to assess the possibility of difficult intubation before MDO.

At present, mouth opening degree, head and neck activity, thyromental distance, ratio of thyromental height to distance, and Mallampati classification are used to assess the possibility of difficult intubation among the general surgical population [12, 13]. However, these prediction methods often lack standard data for children, especially for infants, so at present there is no prediction method that can be reliably applied to RS patients. A new method to predict intubation difficulty before MDO for RS could reduce perioperative complications and improve clinical outcome.

Cone-beam computed tomography (CBCT) allows for extensive anatomic characterization while avoiding excessive radiation exposure [14, 15]. At present, craniofacial CBCT is routinely used to determine the location of upper airway obstruction and depict the mandibular anatomy of infants with RS under consideration for surgical intervention [16–19]. In this retrospective study, we identified quantitative parameters derived from CBCT images that differed between RS patients with normal or difficult intubation and tested their predictive efficacies by receiving operating characteristic (ROC) analyses. These analyses identified three such parameters that distinguish normal from difficult intubation prior to MDO for RS patients with high sensitivity and predictive value.

## Methods

This was a retrospective study and was granted permission to access and use these medical records by the ethics committee of Guangzhou Women and Children’s Medical Center .

Our multidisciplinary team followed a comprehensive algorithm using physical examination, laboratory, endoscopic, and polysomnography findings to assess the severity of airway obstruction. Exclusion criteria were (1) severe cardiopulmonary disease, (2) head and neck tumors or trauma leading to local anatomical structure changes, (3) laryngomalacia, brain-induced central apnea, or mixed apnea, and (4) other anomalies unrelated to RS causing airway obstruction.

All patients underwent intubation by the same experienced anesthesiologist. Patients were divided into two groups according to the Cormack−Lehane classification recorded in the anesthesia record. The degree of difficult intubation was graded as follows: grade I, glottis was completely exposed; grade II, glottis was partially exposed; grade III, epiglottis only was exposed; grade IV, glottis and epiglottis were not seen by endoscopy. Patients of grade I/II were defined as the normal intubation group (group A), while those of grade III/IV were defined as the difficult intubation group (group B). Among infants in the two groups, baseline characteristics collected were sex, age, gestational age, and body mass index (BMI).

CBCT measurements.

**Fig. 1:**
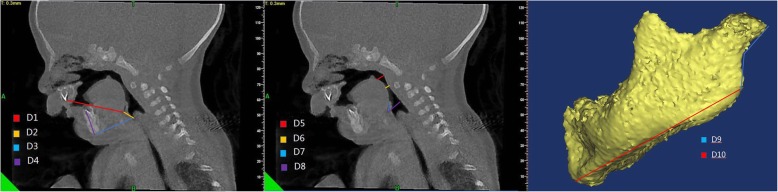
Upper airway distances D1–D11 derived from 3D reconstructions of craniofacial CBCT images acquired prior to mandibular distention osteogenesis for treatment of Robin sequence. Distances D1 to D10 are shown while D11 is the sum of D9 plus D10

**Fig. 2:**
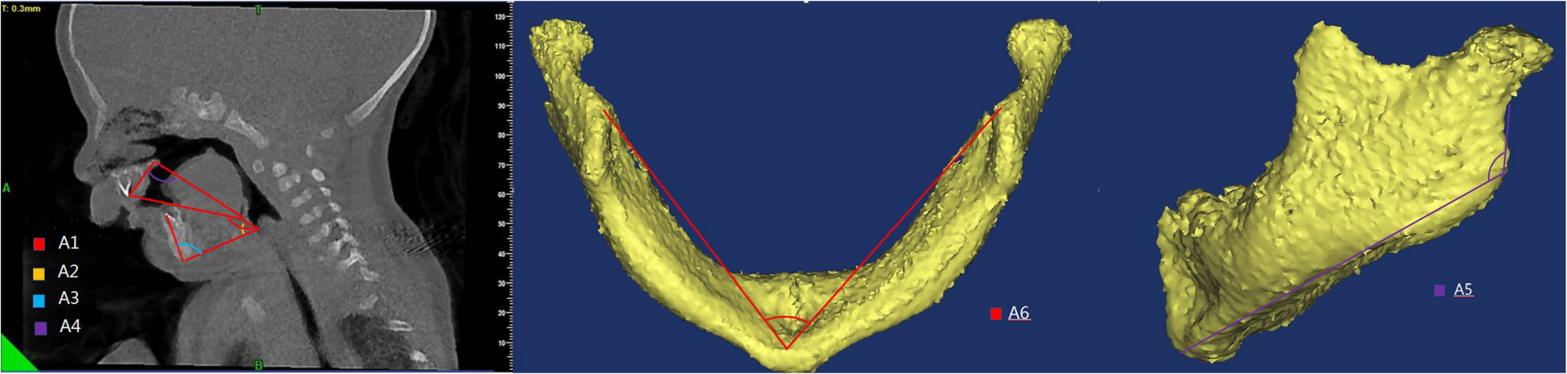
Measurements of upper airway angles A1 to A6

**Fig. 3:**
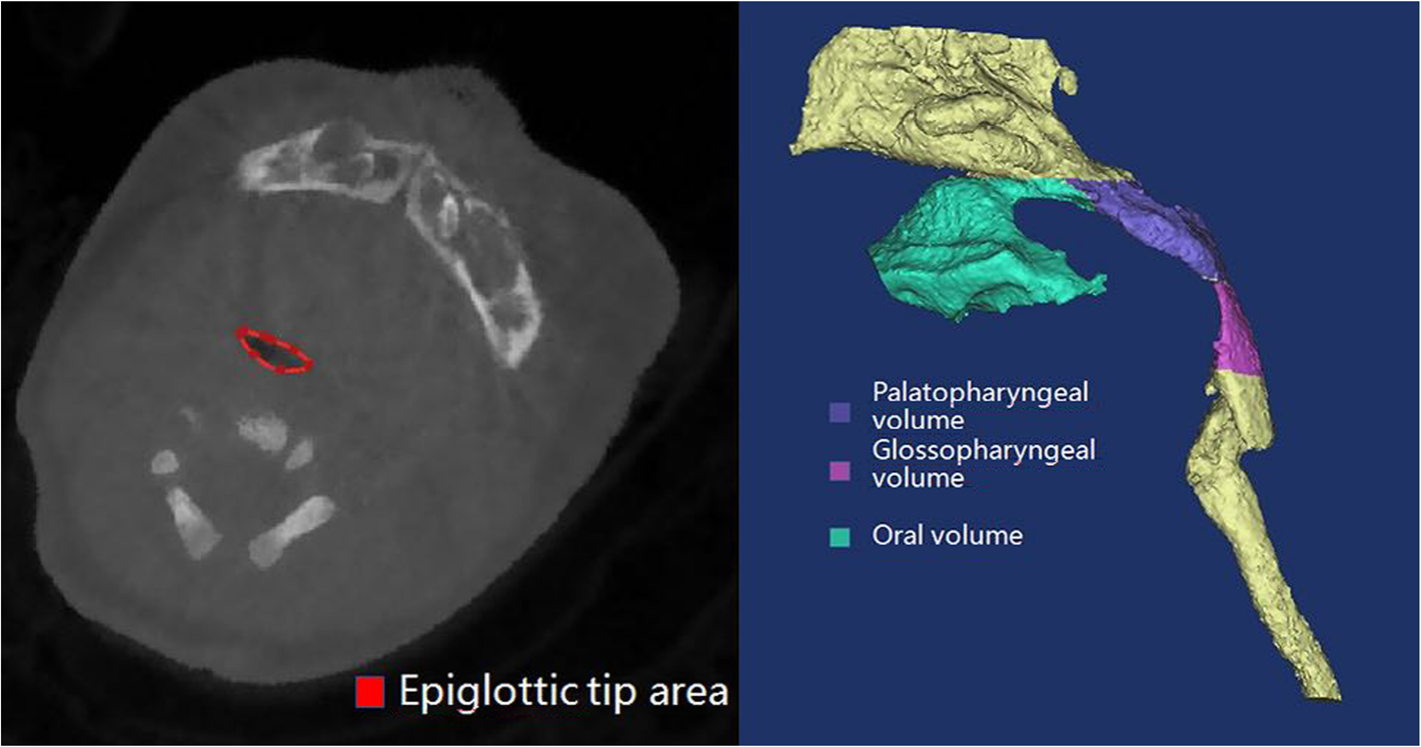
Measurements of upper airway cross-sectional area and segment volumes

Cone-beam CT scans were obtained as part of clinical management using standard institutional protocols. All images were acquired with patients in the left-lateral position at slice thickness between 0.625 mm and 1.25 mm. Axial images were reformatted parallel to the Frankfort horizontal plane and sagittal images were subsequently generated, providing a standardized reference plane. Two experienced raters performed CT analysis for all patients. All CT reformatting and analyses were conducted using MIMICS 17.0 image processing software (Materialise NV, Leuven, Belgium). Airway volumes for each division were calculated on axial images using region of interest (ROI) analysis set at a threshold for air density and the MIMICS ROI volume calculator. Volumes occupied by the radio-opaque border of an artificial airway were not included in the reported palatine pharyngeal volume and glossopharyngeal volume. Craniocaudal lengths for each division were calculated on the reformatted sagittal images. Mandible measures were performed using 3D reconstructed views. A total of 21 parameters (Table 1) were measured as potential predictors of difficult tracheal intubation by a special surveyor. Each index was measured three times by an experienced rater and the average value was taken as the final result. An additional rater performed a second reading to evaluate inter-rater reliability. These parameters included eleven distances (D1 − D11) (Fig. 1), six angles (A1 − A6) (Fig. 2), one airway cross-sectional area, and three volumes (Fig. 3).

**Table 1:** Definition of all CT Measurements

CT Measurements	Definition of all CT Measurements
D1	Distance between the upper central alveolar ridge and root of the epiglottis
D2	Distance between the root of the epiglottis and glottis midpoint
D3	Distance between the end of the mandible and glottis midpoint
D4	Height of the mandible
D5	Distance between the uvula and posterior pharyngeal wall
D6	Distance between the root of the tongue and posterior pharyngeal wall
D7	Distance between the epiglottis and posterior pharyngeal wall
D8	Length of the epiglottis
D9	Length of the mandibular ramus
D10	Length of the mandible body
D11	Length of the mandible
A1	Angle between lines D1 and D2
A2	The angle between line D2 and the lower edge of the upper central alveolar ridge to the glottis midpoint
A3	The angle of lines D3 and D4
A4	The angle of the point of the lower edge of the upper central alveolar ridge to the trailing edge of the hard palate and then to the root of epiglottis
A5	The angle of the mandible
A6	The angle of the gonion to the angle of the mandible
Airway section area at the tip of epiglottis	The airway section area at the tip of epiglottis
Oral volume	Mouth volume from upper and lower alveolar ridge to the posterior edge of the hard palate
Palatine pharyngeal volume	Palatine pharyngeal volume from the posterior border of the hard palate to the edge of the soft palate
Glossopharyngeal volume	Glossopharyngeal volume from soft palate palatal cusp to epiglottis upper edge.

*D* Distance, *A* Angle

### Statistical analyses.

All statistical analyses were performed using SPSS21.0 (IBM, Armonk, NY, USA). To control for differences in skeletal distance among patients of various sizes and ages, all distances were normalized to each patient’s nasion to sella turcica center distance according to the formula y^(norm)^ = y/y^NB^, where y is the raw measure and y^NB^ is the nasion to sella turcica center distance. Baseline clinicodemographic characteristics of the two RS patient groups were compared by t test, while CT measurements were compared by the Mann-Whitney rank sum test. A *P* < 0.05 (two-tailed) was considered significant for all tests. Spearman’s rank correlation coefficient (ρ) was used to evaluate inter-rater reliability respectively, with *ρ* > 0.9 indicating high reliability. According to the test results, a clinical prediction rule was established. Thirty-two individual patient datasets were randomly selected as training sets to build the decision tree model, and the remaining 37 datasets were used as a prediction set to verify the prediction rule. A receiving operating characteristic (ROC) curve was constructed to evaluate predictive efficacy.

## Results

Baseline characteristics of normal and difficult intubation groups of RS patients.

Of the 69 patients enrolled, 30 were classified as normal intubation cases (group A) and 39 as difficult intubation cases (group B), for an overall difficult intubation incidence of 56.52% (Group B/total). There was no significant difference in sex ratio, weight, height, BMI, or gestational age between groups (*P* > 0.05) (Table 2).

**Table 2:** Baseline characteristic of the two groups of RS patients

Variable	Normal intubation group(*n* = 30)	Difficult intubation group(*n* = 39)	*P* Value
Female, No. (%)	12 (40.0)	14 (35.9)	0.922
Birth weight.kg. (mean (sd))	3.01 (0.56)	2.97 (0.58)	0.762
Weight.kg. (mean (sd))	3.70 (0.79)	3.46 (0.58)	0.14
Birth height.cm. (mean (sd))	50.70 (2.25)	49.67 (3.28)	0.232
Height.cm. (mean (sd))	52.03 (3.42)	51.19 (3.44)	0.32
Gestational age. Week. (mean (sd))	38.22 (2.39)	38.48 (1.78)	0.611
BMI (mean (sd))	13.52 (1.87)	12.98 (1.77)	0.233

A comparison of baseline characteristic between these groups can be found in the Table. P<0.05 means a significant difference between the two groups

### Comparison of CBCT measures between groups.

The inter-rater reliability of CBCT parameters met the requirement of *ρ* > 0.9. The distance between the root of the tongue and posterior pharyngeal wall (D6) was significantly shorter, the bilateral mandibular angle (A5) shallower, and the cross-sectional area at the epiglottis tip smaller in the difficult intubation group (all *P* < 0.05) (Table 3).

**Table 3:** Reliability and Comparison of Upper Airway CT Measures between Groups

Variable	Normal intubation group(*n* = 30)	Difficult intubation group(*n* = 39)	*ρ* Value	*P* Value
D1 (mean (sd))	40.86 (3.15)	39.84 (2.80)	0.997	0.192
D2 (mean (sd))	6.92 (3.11)	6.28 (2.09)	0.991	0.357
D3 (mean (sd))	25.02 (3.96)	23.44 (4.31)	0.998	0.164
D4 (mean (sd))	15.52 (2.09)	15.63 (3.17)	0.993	0.877
D5 (mean (sd))	3.79 (1.44)	3.69 (1.33)	0.994	0.772
D6 (mean (sd))	2.78 (1.65)	2.00 (1.19)	0.997	0.045^*^
D7 (mean (sd))	4.87 (2.29)	5.27 (2.18)	0.999	0.511
D8 (mean (sd))	5.66 (1.77)	5.63 (0.99)	0.994	0.942
D9. Left. (mean (sd))	16.54 (2.55)	15.32 (2.49)	0.998	0.068
D9.Right. (mean (sd))	16.41 (2.50)	15.19 (2.67)	0.998	0.078
D10.Left. (mean (sd))	40.32 (3.83)	40.04 (3.05)	0.999	0.762
D10.Right. (mean (sd))	40.54 (4.01)	40.08 (2.95)	0.997	0.613
D11.Left. (mean (sd))	56.91 (5.58)	55.41 (3.77)	0.997	0.227
D11.Right. (mean (sd))	54.96 (11.14)	53.87 (9.58)	0.993	0.686
Area (mean (sd))	40.64 (19.34)	25.61 (11.72)	0.995	0.001^*^
A1 (mean (sd))	144.29 (14.25)	142.53 (19.61)	0.999	0.705
A2 (mean (sd))	4.82 (2.26)	4.63 (2.35)	0.998	0.758
A3 (mean (sd))	116.71 (11.20)	118.45 (14.62)	0.999	0.622
A4 (mean (sd))	100.05 (12.54)	101.60 (7.18)	0.998	0.554
A5.Left. (mean (sd))	135.46 (4.89)	131.45 (8.12)	0.997	0.012^*^
A5.Right. (mean (sd))	135.74 (6.18)	130.73 (8.00)	0.998	0.01^*^
A6 (mean (sd))	87.22 (7.78)	88.08 (8.14)	0.999	0.675
Oral volume (mean (sd))	2000.66 (1389.55)	1948.27 (1192.55)	0.992	0.88
Palatine pharyngeal volume (mean (sd))	652.45 (410.90)	538.90 (267.63)	0.993	0.212
Glossopharyngeal volume (mean (sd))	400.81 (226.37)	321.45 (274.75)	0.992	0.239

Spearman’s rank correlation coefficient was used to evaluate the Inter-observer correlation

*D* Distance, *A* Angle Area: Airway section area at the tip of epiglottis

*Statistically significant at *p* < 0.05

*P*<0.05 means a significant difference between the two groups

ρ>0.9 shows that the measurement results are credible

### Construction of a clinical prediction rule

According to the test results, D6, A5, and cross-sectional area at the epiglottis tip differed significantly between normal and difficult intubation groups. However, the measurement of D6 is based on soft tissue images and so can be influenced by tongue movement, which is not conducive to clinical application. At the same time, not all hospitals have the capacity for three-dimensional reconstruction of CT images, so A5 is not widely applicable. Alternatively, it may be possible to use radiation-free methods such as magnetic resonance imaging (MRI) to measure the cross-sectional area at the epiglottis tip. Considering these factors, we constructed a decision tree model by the airway cross-sectional area at the epiglottis tip (Fig. 3) using Classification and Regression Trees (CART) for predicting difficult intubation. When the cross-sectional area was more than 36.97 mm^2^, difficult intubation was more likely, while normal intubation was more likely when the cross-sectional area was less than 36.97 mm^2^.

### Evaluation of the decision tree model

**Fig. 4:**
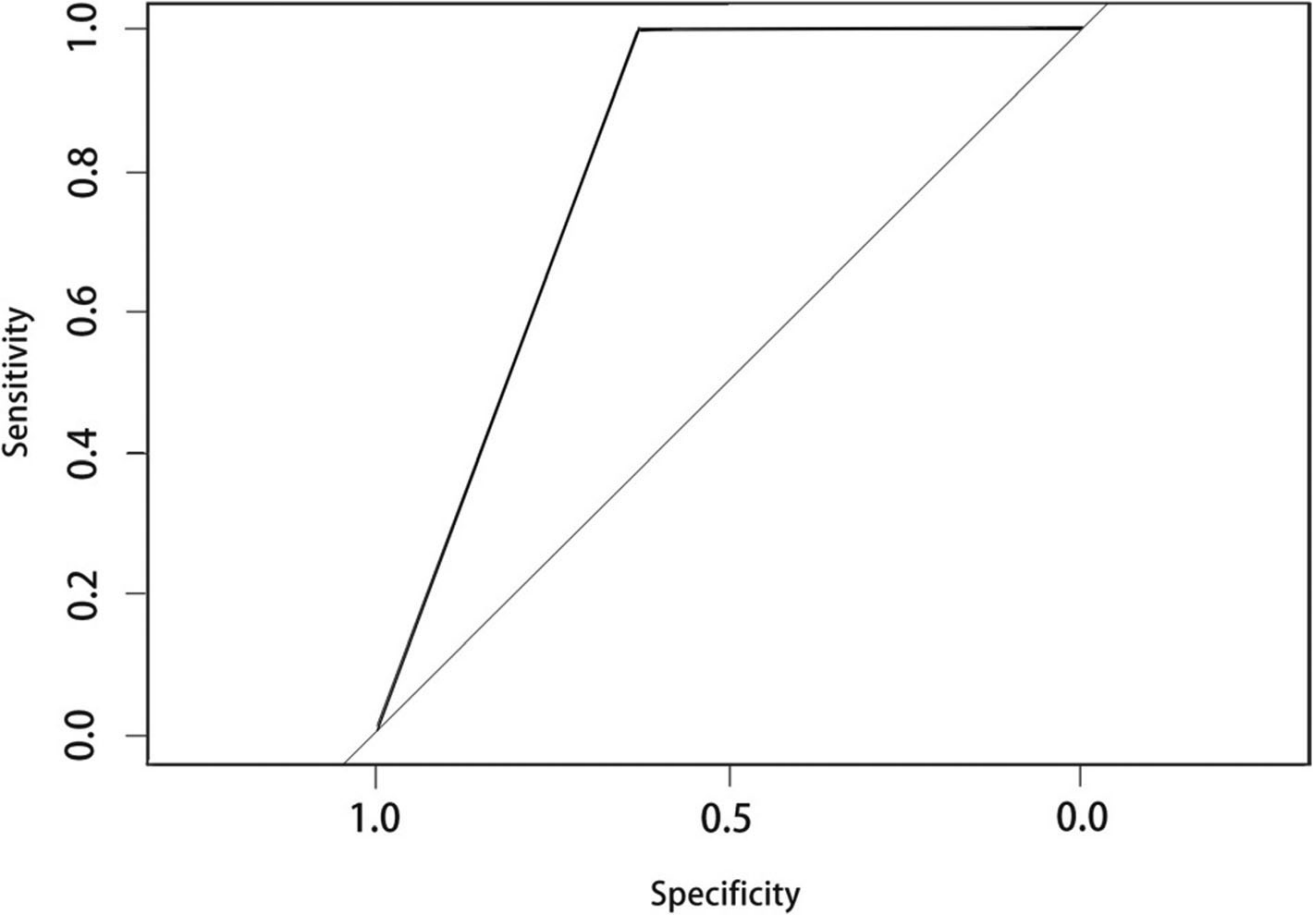
Receiving operating characteristic (ROC) curve used to evaluate the efficacy of the prediction rule based on epiglottis tip cross-sectional area

Based on CART evaluation, the airway cross-sectional area at the epiglottis tip was subjected to ROC analysis, which yielded an area under of ROC curve 0.8125 (Fig. 4) and prediction of difficult intubation with 100% sensitivity, 62.5% specificity, 78.6% positive predictive value, and 100% negative predictive value (Table 4).

**Table 4:** Results of the decision tree model for the prediction set

Sensitivity	Specificity	Accuracy	Positive predictive value	negative predictive value	AUC
(%)	(%)	(%)	(%)	(%)	
1	62.5	78.571	66.667	1	0.8125

## Discussion

This study compared multiple airway dimensions from CT images between RS patients demonstrating normal or difficult intubation during MDO to identify factors useful for presurgical prediction of difficult airway management. Over half of this patient cohort exhibited difficult intubation, and such patients demonstrated a shorter distance between the root of the tongue and posterior pharyngeal wall (D6), a shallower bilateral mandibular angle (A5), and smaller cross-sectional area at the epiglottis tip (Table 3). Based on these findings, we established a clinical prediction rule and verified its efficacy by ROC curve analysis. While tongue root to posterior pharyngeal wall distance (D6) differed significantly between groups, it is also influenced by tongue movement and so may not be reliable for clinical applications. Similarly, many hospitals lack the technology for routine three-dimensional reconstruction of CT images, limiting the use of A5. Therefore, in an attempt to simplify the CT composite score for routine clinical use, we constructed a decision tree model based only one cross-sectional area at the epiglottis tip (Fig. 3) as this metric is not influenced by tongue movement and may be measurable using radiation-free techniques, such as MRI. ROC analysis of this parameter yielded a high AUC (0.8125) using a cut-off cross-sectional area of 36.97 mm^2^, indicating that a cross-sectional area above 36.97 mm^2^ is predictive of difficult intubation.

Mallampati score, nail−chin spacing, chest−chin spacing, upper and lower incisor spacing, mandibular protrusion, cervical retroversion, and ratio of thyromental height to distance are the most widely used methods to identify laryngoscopic exposure difficulties [20–25]. However, most of these methods were established by screening the general population, and are not applicable for patients with maxillofacial deformities [26]. Robin sequence patients have unusual and highly heterogeneous jaw and upper airway morphologies, making it difficult to predict difficult intubation. Computed tomography can be used to evaluate infant bony and soft tissue anatomy of the upper airway in 2 and 3 dimensions, which is not possible with cephalometrics [27–29]. While CT scanning does require radiation exposure, maxillofacial CT is a routine preoperative examination for MDO [16–18], so this evaluation method will not require additional exposure. Further, cone-beam delivery can markedly reduce total radiation dose, so there is no additional safety limitation for clinical practice. Surgical treatment is often unavoidable for the treatment of severe RS [19], and early identification of difficult intubation will help reduce complications from multiple intubation attempts.

This is an exploratory study and has several limitations. First, we were unable to observe the effects of mouth opening on glottic exposure in children with oral closure and quiet breathing during CT scan. The small sample size also limits statistical strength, so other factors predictive of difficult intubation may have been missed. However, we did try to minimize the impact of growth, development, and age through normalization of the CT metrics to baseline values. In addition, this study was conducted at a single center, which may introduce selection bias. For instance, these CBCT metrics were derived from RS infants with severe airway obstruction, and it is not clear whether they persist in infants with mild airway obstruction. However, only severe RS patients require presurgical intubation, so we believe that patient selection does not limit the clinical applicability of the prediction rule. Severe RS patients who need MDO all have potentially life-threatening breathing difficulties. In order to minimize the risk of airway obstruction, our hospital stipulates no more than two attempts at laryngoscopic visualization and intubation. Therefore, we have no clinical information on patients with three or more unsuccessful intubation attempts. This is why patients were divided into normal and difficult intubation groups according to Cormack−Lehane classification instead of by the number of laryngoscopic visualization and intubation attempts.

This work represents a first step toward development of an evidence-based decision tool for predicting difficult intubation in patients with RS, but prospective validation is needed. To further advance our understanding of factors conferring difficult intubation in children with RS, we plan to compare other airway and bone measurements as well as clinical severity measurements. Future work should also assess the effectiveness of imaging modalities that do not involve ionizing radiation, such as MRI.

## Conclusion

Computed tomography was used to quantify morphological parameters of the upper airway predictive of difficult intubation during mandibular distraction osteogenesis for infants with Robin sequence. These measures may help guide RS treatment decisions.

## Data Availability

All data generated or analyzed during this study are included in this published article. The original data can be viewed on the website: (http://www.chictr.org.cn/index.aspx, Registration No. ChiCTR1800018252, NaZhang, 7 Sept 2018).

## Abbreviations

AUC: Area under curve
BMI: Body mass index
CART: Classification and Regression Tree
CBCT: Cone-beam computed tomography
CT: Computed tomography
MDO: Mandibular distraction osteogenesis
ROC: Receiver operating characteristic
ROI: Region of interest
RS: Robin sequence
TBAO: Tongue-based airway obstruction
